# Multi-Template Mesiotemporal Lobe Segmentation: Effects of Surface and Volume Feature Modeling

**DOI:** 10.3389/fninf.2018.00039

**Published:** 2018-07-12

**Authors:** Hosung Kim, Benoit Caldairou, Andrea Bernasconi, Neda Bernasconi

**Affiliations:** ^1^Neuroimaging of Epilepsy Laboratory, McConnell Brain Imaging Center, Montreal Neurological Institute and Hospital, McGill University, Montreal, QC, Canada; ^2^Laboratory of Neuro Imaging, Department of Neurology, Stevens Neuroimaging and Informatics Institute, University of Southern California, Los Angeles, CA, United States

**Keywords:** label fusion, multiatlas segmentation, surface feature modeling, medial temporal lobe (MTL), epilepsy, temporal Lobe

## Abstract

Numerous neurological disorders are associated with atrophy of mesiotemporal lobe structures, including the hippocampus (HP), amygdala (AM), and entorhinal cortex (EC). Accurate segmentation of these structures is, therefore, necessary for understanding the disease process and patient management. Recent multiple-template segmentation algorithms have shown excellent performance in HP segmentation. Purely surface-based methods precisely describe structural boundary but their performance likely depends on a large template library, as segmentation suffers when the boundaries of template and individual MRI are not well aligned while volume-based methods are less dependent. So far only few algorithms attempted segmentation of entire mesiotemporal structures including the parahippocampus. We compared performance of surface- and volume-based approaches in segmenting the three mesiotemporal structures and assess the effects of different environments (i.e., size of templates, under pathology). We also proposed an algorithm that combined surface- with volume-derived similarity measures for optimal template selection. To further improve the method, we introduced two new modules: (1) a non-linear registration that is driven by volume-based intensities and features sampled on deformable template surfaces; (2) a shape averaging based on regional weighting using multi-scale global-to-local icosahedron sampling. Compared to manual segmentations, our approach, namely *HybridMulti* showed high accuracy in 40 healthy controls (mean Dice index for HP/AM/EC = 89.7/89.3/82.9%) and 135 patients with temporal lobe epilepsy (88.7/89.0/82.6%). This accuracy was comparable across two different datasets of 1.5T and 3T MRI. It resulted in the best performance among tested multi-template methods that were either based on volume or surface data alone in terms of accuracy and sensitivity to detect atrophy related to epilepsy. Moreover, unlike purely surface-based multi-template segmentation, *HybridMulti* could maintain accurate performance even with a 50% template library size.

## Introduction

Mesiotemporal lobe (MTL) structures, such as the hippocampus (HP), amygdala (AM), and entorhinal cortex (EC), undergo marked morphological changes in numerous neurological and neuropsychiatric conditions (Wang et al., 2010; Cavedo et al., 2011; Bernhardt et al., 2013; Shi et al., 2013; Joo et al., 2014; Maccotta et al., 2015; Arnone et al., 2016). MRI volumetry has been the most commonly employed technique to assess MTL pathology *in vivo* (Goncharova et al., 2001; Bernasconi et al., 2003). In temporal lobe epilepsy (TLE), the most common surgically-amenable epilepsy in adults, manual MRI volumetry allows defining the side of mesiotemporal atrophy in up to 70–90% of patients (Schramm and Clusmann, 2008), and thereby help identifying the surgical target.

Manual MTL volumetry is a labor-intensive task with high demands on neuroanatomical expertise. Although existing automatic segmentation algorithms produce excellent segmentation results for HP and AM in healthy controls (Collins and Pruessner, 2010), their performance in TLE is challenged by the combined effects of atrophy and positional abnormalities (Kim et al., 2012a). Only a relatively small number of studies have attempted segmentation of the entire MTL regions including parahippocampal gyrus (PHG) (Heckemann et al., 2006; Keihaninejad et al., 2012). A study (Hu et al., 2014) specifically segmented the EC, a PHG subregion considered a core epileptogenic zone in TLE (Bernasconi et al., 2003) with suboptimal accuracy (Dice index = 73%), likely due to challenges imposed by its complex and variable shape.

Volume-based multi-template and label fusion approaches have been designed to account for shape complexity and anatomical variability by selecting a subset of templates from a large library that best describes the target structure (Collins and Pruessner, 2010; Khan et al., 2011). More recently, our previously proposed surface-based SurfMulti method automatically segmented HP using vertex-wise texture and shape sampling (Kim et al., 2012b), demonstrating improved performances compared to purely volumetric techniques (Collins and Pruessner, 2010). However, performance of purely surface-based approaches likely depends on the availability of a large library, as it may be negatively impacted when the boundaries of the template and individual MRI are not well aligned. The label fusion in volume-based approaches has become sophisticated using local weighted averaging (Artaechevarria et al., 2009; Coupé et al., 2011; Eskildsen et al., 2012; Wang et al., 2013; Awate and Whitaker, 2014). These approaches have demonstrated the improvement of segmentation.

MICCAI Grand Challenge on Multiatlas Labeling (Landman and Warfield, 2012) systemically evaluated various multi-template approaches for the segmentation of numerous brain structures but the parahippocampal gyrus. A total of 25 algorithms that were trained by 15 atlases were tested on 20 images. The performance for the hippocampus and the amygdala ranged 82–87 and 75–83% in mean Dice similarity index, respectively. Among the methods that were evaluated, the ones that displayed higher accuracy were the joint label fusion technique that used a joint probability of selected atlases to correct for the bias due to the inclusion of similar atlases in the template library or the training-set (Wang et al., 2013) and the Non-Local STAPLE algorithm that combined Staple method with the non-local means estimator (Asman and Landman, 2013).

The current work aimed at segmenting simultaneously HP, AM, and EC using a large template library (*n* = 175) which included shape and volume variants in relation to TLE (*n* = 135). We tested well-established volume-based and surface-based approaches as well as looked for a possibility of the combined approach. The proposed algorithm, *HybridMulti*, combined surface-based with volume-based similarity measures for optimal template selection. The SurfMulti was based on the linear alignment between the template and individual MRI. Volume-based approaches (Asman and Landman, 2013; Wang et al., 2013) rely also on the accuracy of the linear and non-linear registration. To improve alignment, we introduced a non-linear registration step that incorporates a novel hybrid cost function based on surface and volume. Our algorithm furthermore included a new multi-level feature weighting for shape averaging. We compared MTL segmentation of *HybridMulti* to our previous SurfMulti (Kim et al., 2012b) and two volume-based approaches with/without local weighted averaging (Collins and Pruessner, 2010; Wang et al., 2013); evaluations also took into account the influence of template library size on segmentation performance.

## Methods

**Figure 1:**
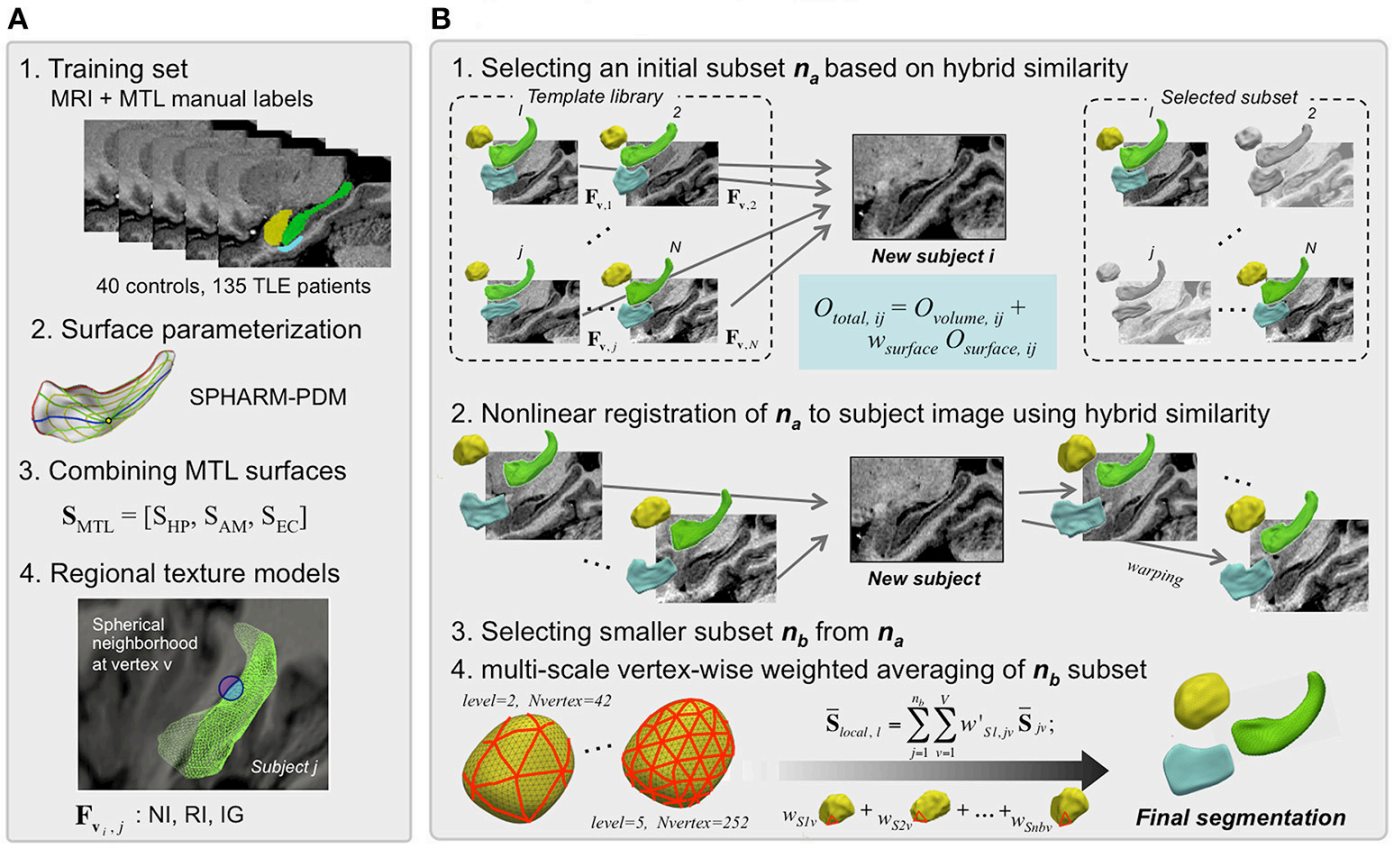
HybridMulti automatic hippocampal segmentation steps. Flowchart of the proposed algorithm (in **A**, steps 2 and 4 are illustrated only for the HP). The segmentation procedure consists mainly of two: template library construction and automated segmentation of mesiotemporal structures. **(A)** Template library construction. **(B)** Automatic segmentation of MTL structures.

**Figure 2:**
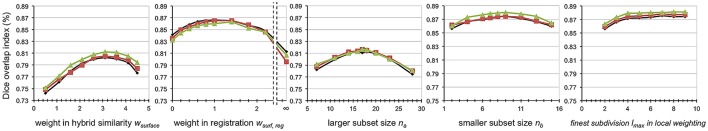
Parameter optimization. All parameters were selected resulting in the best accuracy. The accuracy was measured using mean Dice index based on the three mesiotemporal structures and on three different test-sets (black, red, green) using a three-fold cross validation.

**Figure 3:**
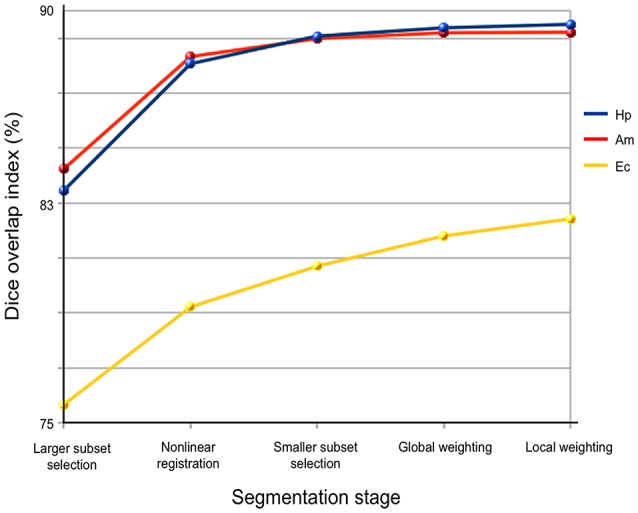
Performance of each processing stage in *HybridMulti*. The Accuracy is evaluated using Dice index.

**Figure 4:**
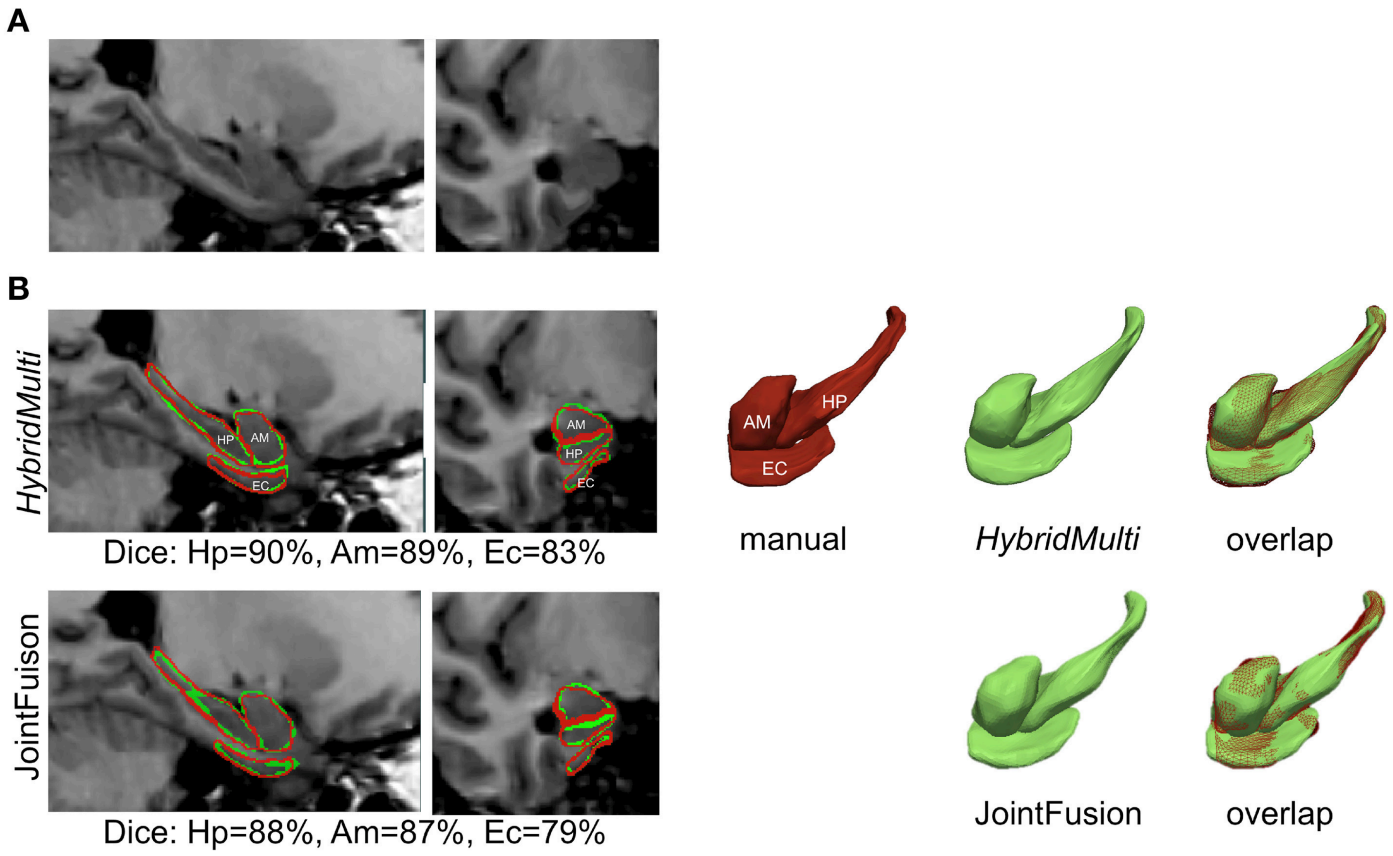
Segmentation of mesiotemporal lobe structures in a patient with atrophy. Shown are overlaps between two best algorithms (*HybridMulti*, JointFusion—green) and manual label (red). **(A)** MRI **(B)** Segmentations overlaid on MRI and in 3D rendering.

*HybridMulti* includes a “template library construction” where the algorithm learns image features using a training-set and an “automatic segmentation” step where the algorithm segments MTL structures for an individual test MRI (Figure 1). Training set consists of MR images and manual labels of controls and patients (Figure 1A). Labels are converted into surface meshes using spherical harmonics and point distribution model (SPHARM-PDM) that ensure shape-inherent point-wise correspondences across subjects (Styner et al., 2004, 2006b). Each surface is mapped onto its corresponding MRI. In the beginning of the segmentation step, the pair of each template image and its MTL surface are mapped on the test image. As the test image does not have its own surface, the surface features extracted on the test image are from the surface of each template. By comparing the features extracted from each template and those from the test image, Surface- with volume-derived similarity measures for optimal template selection are then computed to select an optimal subset *n*_*a*_ (Figure 1B-1). Next, a non-linear registration that is driven by volume-based intensities and features sampled on evolving template surfaces is performed to improve alignment between each template in the subset *n*_*a*_ and the individual MRI (Figure 1B-2). The motivation of using this hybrid registration was to improve the boundary fitting by weighting the features extracted using deformable surfaces as well as to use a consistent similarity measurement in all the steps. After choosing a smaller subset *n*_*b*_, templates are then averaged using adaptive weighting combined with local averaging, which creates the final segmentation (Figure 1B-3,4). The test image's features are updated during the series of the steps including template selection, non-linear registration and weighted averaging as the image and the surface deform. In this manner, the similarity of the deformable surface and the target MTL border is expected to increase and the surface gets a similar shape to the true MTL boundary.

### Template library construction (Figure 1A)

Prior to the subsequent procedures, all MR images in the training-set and the test-set are spatially normalized by registering them into MNI ICBM 152 space. We create a template library that aggregates surface-based regional texture models of HP, AM, and EC as a joint representation of the three MTL structures.

Manually delineated labels of each MTL structure [linearly registered to MNI ICBM-152 space (Collins et al., 1994)] are converted into surface meshes and parameterized using the spherical harmonics and uniform icosahedron-subdivision model (SPHARM-PDM) that guarantees shape-inherent vertex-wise correspondence across subjects (Styner et al., 2006a). MTL surfaces are treated as one concatenated surface, **S**_MTL_ = [S_HP_, S_AM_, S_EC_].

Each surface **S**_MTL_ is mapped to its corresponding MRI. At a given surface vertex ***v***, we define three spherical neighborhoods of 3, 5, and 7 mm radius. These spheres are subdivided into an inner region (IR) and outer region (OR) with respect to the surface boundary, where we compute the following texture features (Kim et al., 2012b): *i) Normalized intensity (NI)*: the ratio between mean intensity and intensity standard deviation for each of IR/OR to capture regional tissue homogeneity. We defined NI_*IR*_, _*i*_ = μ_*IR*_, _*i*_ / SD_*IR*_, _*i*_ and NI_*OR*_, _*i*_ = μ_*OR*_, _*j*_ / SD_*OR*_, _i._; *ii) Relative intensity (RI)*: the ratio of mean intensity between IR and OR to assess the contrast between IR and OR voxels. RI was defined as RI _*i*_ = 2 × (μ_*OR*_, _*i*_ - μ_*IR*__*i*_) / (μ_*OR*_, _*i*_ + μ_*IR*_, _*i*_); *iii) Intensity gradient (IG)*: the 1st derivative of intensity along x-, y-, and z-directions to capture edge information was summarized into the magnitude as IG = $\sqrt{g_{x}^{2}+g_{y}^{2}+g_{z}^{2}}=\sqrt{\frac{\partial I}{\partial x}+\frac{\partial I}{\partial y}+\frac{\partial I}{\partial z}}$. [*x y z*] is a voxel location and I is an image.

These texture features comprises a set of “true” feature vectors (3 normalized intensity + 3 relatively intensity + 3 gradients = 9 features), **F**_v__, j_ extracted at **v**-th vertex on the *j*^*th*^ (1 … *j … N)* surface template. Previously we demonstrated that each feature almost equally contributed to the segmentation accuracy and observed the optimal result using all the features. Notably, we did not use the shape features proposed in our previous surface-based framework (Kim et al., 2012b), which was used to constrain the shape deformation in the Automatic segmentation step. The deformation in the current study is instead governed directly by a volume-based non-linear registration (see section Boundary-Weighted Non-linear Registration of Template Subset to Test MRI).

### Automatic segmentation (Figure 1B)

#### Initial template subset selection

From the template library, we first select a subset of candidates that are most similar to the test image. To that end, we compute the hybrid similarity *O*_*total*_ that combined surface-based (*O*_*surface*_) and volume-based (*O*_*volume*_) similarity term between each template *j* and the test MRI *i* using:

(1) $${\text{O}}_{\text{total},\text{ij}}{\;=\text{ O}}_{\text{volume},\text{ ij}}{\;+\text{ w}}_{\text{surface}}{\text{O}}_{\text{surface},\text{ij}}$$

w_*surface*_ is a weighting constant. The surface-based similarity *O*_*surface*_ is defined as:

(2) $$O_{surface,ij}=\text{​}-\text{​}\sum_{\text{v}}\frac{\left\|F_{v,j}\text{​}-\text{​}{\hat{F}}_{v,ij}\right\|}{\sqrt{\frac{1}{N}\sum_{k=1}^{N}{\left(F_{v,k}\text{​}-\text{​}{\bar{F}}_{v}\right)}^{2}}},\;{\bar{F}}_{v}\text{​}=\text{​}\frac{1}{N}\sum_{k=1}^{N}F_{v,k}$$

*O*_*surface*_ is calculated across all surface vertices **v**. It represents a normalized similarity between *true* features extracted from the *j*^*th*^ (1 … *j … N)* template (**F**_v__, j_) and *estimated* features extracted from the test MRI *i* (${\hat{\text{F}}}_{\text{v},i}$)*. O*_*volume*_ can be any similarity function including the cross-correlation or the normalized mutual information (NMI) that quantifies statistical intensity distribution dependency of two images *A* and *B* (Studholme et al., 1999). The computation of cross-correlation is generally faster while the NMI is more robust in similarity of multi-modal images compared to each other. For computational efficiency, we compute *O*_*volume*_ within a mask defined by dilating the current template label three times. The number of selected templates (*n*_*a*_) was empirically determined to maximize *O*_*total*_ (see section Parameter Selection).

#### Boundary-weighted non-linear registration of template subset to test MRI

Each template MRI is non-linearly registered to the test MRI to increase shape similarity. To estimate the deformation field from a template **T** to the test MRI **I**, a “conventional” non-linear registration iteratively matches intensity features by maximizing a volume-based similarity function *O*_*vol*_, _*reg*_. Accordingly, the deformation field *d* is estimated as:

(3) $$\vec{d}=\underset{\vec{d}}{\text{arg max}}\;O_{vo,\;reg}(T+\vec{d},I)+O_{smooth}$$

*O*_*smooth*_ is a smoothness term to constrain the estimated deformation. We employed a type of freeform deformation models defined in Collins et al. (1995). To improve the registration accuracy, we increase the weight of voxels on and nearby the target boundary by incorporating a similarity measure derived from the template surface evolving during the registration with the original volume similarity. Let **S**_*MTL*_, _*T*_ be the *true* template surface on the original MRI and **S**_*MTL*_, _*S*_ an *estimated* template mapped onto the test MRI. We define **S**_*MTL*_, _*S*_ by deforming **S**_*MTL*_, _*T*_ using the deformation field estimated at the current iteration. A surface-based feature similarity measure between **S**_*MTL*_, _*T*_ and **S**_*MTL*_, _*S*_ is defined as:

(4) $$\begin{array}{l} O_{surf,\;reg}=-\frac{\sum_{v}\left(F_{v,\;T}-{\bar{F}}_{v,\;T})(F_{v,\;\hat{S}}-{\bar{F}}_{v,\;\hat{S}}\right)}{\sqrt{\sum_{v}{\left(F_{v,\;T}-{\bar{F}}_{v,\;T}\right)}^{2}}\sqrt{\sum_{v}{\left(F_{v,\;\hat{S}}-{\bar{F}}_{v,\;\hat{S}}\right)}^{2}}},\\ \;F_{v}=(\mu_{v,\;OR}-\mu_{v,\;IR})/(\mu_{v,\;OR}+\mu_{v,\;IR}) \end{array}$$

where **v** is a vertex on surfaces **S**; **F**_v_ is the relative intensity defined in **2.1**. Therefore, *O*_*surf*_, _*reg*_ is a correlation coefficient between feature **F**_v_,_*T*_ extracted on **S**_*MTL*_, _*T*_ and feature **F**_v_, _Ŝ_ extracted on **S**_*MTL*_, _Ŝ_. To estimate the deformation field, we redefine the Equation (3) as:

(5) $$\begin{array}{l} \vec{d}=\underset{\vec{d}}{\text{arg max }}O_{hybrid,\;reg}(T+\vec{d},I)+O_{smooth},\\ \;O_{hybrid,\;reg}=O_{vol,\;reg}+w_{surf,\;reg}O_{surf,\;reg} \end{array}$$

*O*_*vol*_, _*reg*_ is the correlation coefficient over a volume of interest (here, a geometric union of all MTL template labels in the library, subsequently dilated 5 times for more extensive spatial coverage) as in Collins and Pruessner (2010). A larger weight *w*_*surf*_, _*reg*_ moves **S**_*MTL*_, _*S*_ more rapidly to areas presenting with feature characteristics similar to those on the surface of the template image. Finally, Equation (5) is optimized using a derivative-free 3D Nelder-Mead Simplex approach (Lagarias et al., 1998) as also known as the simplex method, is a commonly applied approach. This method is applied to non-linear optimization problems for which derivatives may not be known and is robust against the local minima problem. This function has been used as the standard optimization method in the non-linear registration algorithm (Collins et al., 1995) we adopted in the current paper.

#### Subset restriction and global weighed averaging

The non-linear registration in the previous section (Boundary-weighted Non-linear Registration of Template Subset to Test MRI) is applied to decrease shape variability and to increase similarity between the template-subset and test image. From the initially selected *n*_*a*_ template-subset (*n*_*a*_< *N*), we choose an even smaller subset of the *n*_*b*_ most similar templates (*n*_*b*_< *n*_*a*_< *N*) based on Equation (1), increasing computational efficiency in subsequent steps. We determine *n*_*b*_ empirically, which will be evaluated in the section Parameter Optimization.

Optimal global weights for these *n*_*b*_ templates are calculated using the similarity function Equation (2) as in Kim et al. (2012b). Let **w**_S_ and **w**_F_ be *n*_*b*_ × 1 weight vectors for optimal surfaces and features. We then define $\bar{\text{S}}$ as the average surface of the *n*_*b*_ template-subset as:

(6) $${\bar{S}}_{global}\text{​}=\text{​}\sum_{j=1}^{n_{b}}w_{F,j}F_{v,j};\;w_{F}\text{​}=\text{​}\left[w_{F,1},w_{F,2},\ldots ,\right];\;\sum w_{F,j}\text{​}=\text{​}1$$

Analogously, we define the weighted mean and SD of features at a given vertex **v**_i_ by:

(7) $$\begin{array}{l} {\bar{F}}_{v}=\sum_{j=1}^{n_{b}}w_{F,j\;}F_{v,j};\;w_{F}=\left[w_{F,1},\;w_{F,2},\ldots ,w_{F,n_{b}}\right];\;\sum w_{F,j}=1;\; \end{array}$$

(8) $$\begin{array}{l} \sigma_{F,v}=\sqrt{\sum_{j=1}^{n_{b}}w_{F,j\;}{\left(F_{v,j}-{\bar{F}}_{v}\right)}^{2}\;} \end{array}$$

Similarity from Equation (2) can be formulated for the template-subset *n*_*b*_:

(9) $$O_{subset}=-\sum_{\text{v}}\frac{\left\|{\bar{F}}_{v}-{\hat{F}}_{v,\;\bar{s}}\;\right\|}{\sigma_{F,v}}$$

${\hat{\text{F}}}_{v,\;\bar{s}}$ is the *estimated* feature-set computed on the averaged surface $\bar{\text{S}}$ mapped on the test image. In the above formulas, weights are determined by maximizing the similarity between the *n*_*b*_ template-subset and test image.

(10) $$w=[w_{s}\;w_{F}]={\text{arg}}_{w}\text{max }O_{subset}$$

We initialized all components of **w**_S_ and **w**_F_ to 1/n. The cost function *O*_*subset*_ is optimized using the multivariate derivative-free Nelder-Mead approach (Lagarias et al., 1998).

#### Multi-level local weighted averaging

To incorporate a local weighting to Equations (5–9), the resulting surface $\bar{\text{S}}$ in Equation (10) is resampled through icosahedron-subdivision (Styner et al., 2006b), first at the coarsest level *l* = *l*_0_. We determine weights at each sampling vertex, and interpolate these weights to vertices at the next finer level *l*_1_. Let **w**_S__*l*_ be a *n*_*b*_ □ *m* weight matrix: *m* is the number of vertices at level *l*. We compute **w'**_S__*l*_, (a *n*_*b*_ □ *V* vector) by interpolating **w**_S_, _*l*_ to all vertices *v* [*1, 2*, …,*V*] of the original surface [[Inline Image]] (*V* > *m*). For interpolation, we use the Fast Spherical Linear Interpolation (Shoemake, 1985). We define the locally weighted average surface as:

(11) $${\overset{-}{S}}_{local,l}=\sum_{j\;=\;1}^{nb}\overset{v}{\underset{v\;=\;1}{\sum }}{w^{\prime }}_{sl,jv},{\overset{-}{S}}_{jv};\sum_{j\;=\;1}^{nb}\overset{v}{\underset{v\;=\;1}{\sum }}{w^{\prime }}_{sl,jv}=V$$

The similarity function at the level *l* was defined as:

(12) $$O_{subset,l}=\text{​}-\text{​}\sum_{i}\frac{\left\|{\bar{F}}_{v_{i}}\text{​}-\text{​}{\hat{F}}_{v_{i},{\bar{S}}_{local}}\right\|}{\sigma_{F}};\;w_{S\;l}=\text{arg }max_{w}O_{subset}$$

To achieve the final segmentation of all three MTL structures, we optimized **w**_S__*l*_ using the Nelder-Mead method while increasing subdivision level *l* = [*l* | *l*_0_*, l*_1_*,…, l*_*max*_]. The algorithm stops when **Equation** (**11**) stops increasing or *l* reaches preset *l*_*max*_ to prevent from an extensive computation. The proposed multi-level approach using different subdivisions is mainly for coarse-to-fine spatial fitting and the use of this strategy avoids the introduction of a constraint term preventing from local minima while the surface shape gets finer. In the current study, we set the coarsest level (*l*_0_ = 2) where 42 equally distributed vertices are sampled; the finest level *l*_*max*_ is determined empirically (See section MRI Acquisition).

## Experiments and results

### Experiments

#### Subjects

Our training-set included 40 healthy controls (18 men; mean ± SD age = 33 ± 12 years) and 135 drug-resistant TLE patients (61 men; mean ± SD age = 37 ± 11 years). TLE diagnosis and lateralization of the side of the seizure focus into left TLE (*n* = 65) and right TLE (*n* = 70) were determined by a comprehensive evaluation including video-EEG recordings and MRI. The Ethics Committee of the Montreal Neurological Institute and Hospital approved the study and written informed consent was obtained from all participants.

#### MRI acquisition

MR images were acquired on a 1.5 Tesla Phillips Gyroscan using a T1-weighted FFE sequence (TR = 18 ms; TE = 10 ms; NEX = 1; flip angle = 30°; matrix size = 256 □ 256; FOV = 256 mm; slice thickness = 1 mm), yielding 1 mm-isotropic voxels. Images underwent intensity non-uniformity correction (Sled et al., 1998). Intensities were normalized and images were linearly registered to the MNI ICBM-152 template (Collins et al., 1994). MTL structures were manually segmented by an expert using the protocol described in Bernasconi et al. (2003). Based on z-score normalization with respect to volumes in controls, 81 (60%) patients showed hippocampal atrophy (i.e., a z-score below −2) ipsilateral to the seizure focus.

We also acquired 3T T1-weighted images on Siemens Trio Tim scanner using a 32-channel phased-array head coil. T1-weighted images were acquired using 3DMPRAGE with 1 mm isotropic voxels (TR = 3,000 ms, TE = 4.32 ms, TI = 1,500 ms, flip angle = 7°, matrix size = 336 × 384, FOV = 201 × 229 mm). This data was used to evaluate whether the algorithm consistently selected the same or similar parameter values for different dataset. The 3T dataset included 39 healthy controls and 84 drug-resistant TLE patients who were further classified into left TLE (*n* = 38) and right TLE (*n* = 46).

#### Evaluation metrics

To quantify the accuracy of automated segmentations, we computed the Dice similarity index:*D* = 2*xv*(*M*∩*A*)/(*v*(*M*)+*v*(*A*)), where M/A are the voxels comprising manual/automated labels; “M n A” are voxels in the intersection of M and A; v (·) is the volume operator.

#### Parameter optimization

Based on maximal Dice overlap index between automated and manual labels, the following parameters were chosen empirically: weight of surface-based similarity *w*_*surfac*__e_ to select the optimal subset as in Equation (1); weight of surface-based similarity *w*_*surf*_, _*reg*_ used in non-linear registration; size of initial template-subset *n*_*a*_; size of final template-subset *n*_*b*_; and finest subdivision *l*_*max*_ in local weighting. We validated *HybridMulti* using a three-fold cross-validation where we subdivided our data into 3 sets with an almost equal sized sample (*n* = 58,58,59) and merged two sets among them to create a training-set and used the remaining set as a test-set while we balanced the proportion of controls (~25%) and patients (~75%) per set. The optimal parameters that resulted in most accurate segmentation were selected for each training-set. We segmented the test-set based on their corresponding training-set and the parameters. We repeated this process three times while all the three sets were tested.

#### Performance at each segmentation stage

Segmentation accuracy was evaluated at the following stages: *i)* initial *n*_*a*_ template-subset selection; *ii)* non-linear registration; *iii)* final *n*_*b*_ template-subset selection; *iv)* global and local weighted averaging. We compared accuracy at each stage to that of the previous stage using paired *t*-tests.

#### Comparison with state-of-the-art multi-template approaches

We compared Dice indices between *HybridMulti*, and SurfMulti (Kim et al., 2012b), or a volume-based multitemplate approach (VolMulti) based on non-weighted averaging (Collins and Pruessner, 2010) or a volume-based approach (JointFusion) based on local-weighted averaging (Wang et al., 2013) in controls and each patient group using Student's *t*-tests. The parameters for each algorithm were selected empirically (VolMulti: size of subset = 15; JointFusion: search area *r*_s_ = 3 x 3 x 3, patch size *r*_p_ = 3 x 3 x 3, β = 2) which resulted in the best accuracy using a leave-one-out approach.

#### Detection of mesiotemporal atrophy related to the epileptic focus

We assessed the ability of each automatic algorithm to detect each structure's atrophy in TLE groups relative to controls by computing Cohen's d ([mean volume controls—mean volume TLE] / pooled SD) that measures the effect size of a between-group difference, and calculated the significance of the observed effect using *t*-tests.

#### Impact of template library size on segmentation accuracy

Keeping proportions of controls and patients constant, we randomly selected 40 subjects as a test-set. We then created the template library by selecting randomly from the rest of data, with various sizes: *n* = 88 (1/2), *n* = 58 (1/3), *n* = 44 (1/4), and *n* = 35 (1/5) of its original size. We repeated this process 20 times to avoid a possible bias. We evaluated automated segmentation accuracy at these smaller template library sizes.

Significances of all statistical tests were adjusted for multiple comparisons using Bonferroni-correction.

### Results

#### Parameter selection

The parameters resulting in the best segmentation accuracy were selected at very similar values between the 3 test-sets when using a three-fold cross validation. The proposed HybridMulti achieved maximal accuracy with the following parameters: w_surface_ = 3.1, w_surf, reg_ = 1.1, n_a_ = 17, and n_b_ = 8 (average between the 3 test-sets; Figure 2). Use of the cross-correlation or NMI as the similarity function did not make a difference in segmentation accuracy. We thus used the cross-correlation as it was faster to compute. We also found that the local weighting using the finest subdivision l_max_ larger than 5 (producing 252 sampling vertices) maintained the segmentation accuracy without a further improvement. Thus, we chose l_max_ = 5 as a larger l_max_ increased the computational time. JointFusion yielded best results with the following parameters: beta = 0.5; rp = 3; rs = 3. SurfMulti used *n* = 10 for the optimal subset whereas VolMulti used *n* = 14. All the algorithms were tested on a same computing environment (Linux workstation, 1 CPU, 2.30 Ghz, 8 GB RAM). Average computation times per individual hemisphere were 20 or 25 min for HybridMulti (O_volume_ = cross-correlation or NMI, respectively; step-wise: initial subset selection: 1 min; non-linear registration: 10 [cross-correlation] or 15 [NMI] min; smaller subset selection: 0.5 min; global weighting: 3 min; Local weighting: 5.5 min); 15 min VolMulti; 15 min JointFusion; 13 min SurfMulti.

When performing the same evaluation on 3T dataset, we found the parameters yielding the maximal accuracy were selected at very similar values: *w*_*surface*_ = 3.2, *w*_*surf, reg*_ = 1.2, *n*_*a*_ = 17, *n*_*b*_ = 8, and *l*_max_ = 6.

#### Segmentation accuracy in different steps

Accuracy of HybridMulti was improved gradually from the initial selection step and the highest accuracy was achieved at the final local weighted averaging (Figure 3).

Highest improvement was found at the boundary-weighted non-linear registration step for all structures (mean Dice = +4.8%, *p* < 0.0001). Moreover, the proposed non-linear registration that included a surface-term outperformed the original volume-based registration (Collins et al., 1995) (+2.5%, *p* < 0.001). Inclusion of local weighted averaging also significantly improved segmentation of EC (0.7%) and (HP: 0.3%) compared to the global weighting (*p* < 0.05).

#### Performance comparison between algorithms

For all MTL structures, HybridMulti consistently outperformed SurfMulti and VolMulti in patients and controls (*p* < 0.001, Table 1), which was equally significant for 1.5T and 3T data (Table 2). HybridMulti also showed a superior accuracy in TLE patients compared to JointFusion as higher Dice indices were found in HP and EC ipsilaterally and in AM and EC contralaterally (*p* < 0.05). HybridMulti also segmented EC in healthy controls more accurately than JointFusion (*p* < 0.001). This pattern of difference between HybridMulti and JointFusion was similar in 3T data (Table 2).

**Table 1:** Segmentation accuracy using a three-fold cross validation (% mean ± *SD* of Dice similarity index).

	**VolMulti**	**SurfMulti**	**JointFusion**	***HybridMulti***
**CONTROLS**				
HP	84.4 ± 4.1^*^	86.8 ± 2.7^*^	89.5 ± 1.5	89.7 ± 2.1
AM	83.8 ± 4.3^*^	86.3 ± 3.6^*^	88.4 ± 2.8	89.3 ± 2.5
EC	75.8 ± 6.8^*^	79.3 ± 4.6^*^	78.4 ± 4.3^*^	82.9 ± 3.7
**IPSILATERAL**				
HP	80.3 ± 5.4^*^	86.1 ± 3.4^*^	87.3 ± 3.8^*^	88.7 ± 2.5
AM	83.2 ± 4.2^*^	85.2 ± 3.9^*^	88.0 ± 2.9	89.0 ± 2.6
EC	75.2 ± 8.1^*^	77.7 ± 5.2^*^	78.0 ± 6.5^*^	82.6 ± 3.8
**CONTRALATERAL**				
HP	84.0 ± 4.4^*^	86.9 ± 3.1^*^	88.8 ± 3.0	89.4 ± 2.3
AM	83.8 ± 4.2^*^	85.8 ± 3.7^*^	88.3 ± 2.8^*^	89.2 ± 2.7
EC	76.2 ± 7.4^*^	78.8 ± 5.4^*^	78.6 ± 4.8^*^	82.7 ± 4.1

*Ipsilateral/Contralateral refers to the epileptogenic hemisphere. Decreased performance relative to HybridMulti* -

* *: significant after Bonfferoni correction (p < 0.05/36 = 0.0013)*.

**Table 2:** Segmentation accuracy for a smaller set of 3T data (controls: *n* = 39; TLE: *n* = 84) using a three-fold cross validation (% mean ± SD of Dice similarity index).

	**VolMulti**	**SurfMulti**	**JointFusion**	***HybridMulti***
**CONTROLS**				
HP	85.6 ± 3.6^*^	87.3 ± 2.5^*^	89.7 ± 1.4	89.8 ± 1.8
AM	84.3 ± 3.9^*^	86.4 ± 3.1^*^	88.5 ± 2.4	89.4 ± 2.3
EC	77.3 ± 6.4^*^	80.2 ± 4.6^*^	79.1 ± 4.1^*^	83.1 ± 3.3
**IPSILATERAL**				
HP	82.3 ± 5.4^*^	86.1 ± 2.9^*^	87.0 ± 3.6^*^	88.4 ± 2.3
AM	83.2 ± 4.2^*^	84.9 ± 3.8^*^	87.6 ± 2.7^*^	88.9 ± 2.4
EC	76.4 ± 8.2^*^	78.1 ± 4.9^*^	78.8 ± 6.4^*^	82.6 ± 3.5
**CONTRALATERAL**				
HP	84.2 ± 4.3^*^	87.7 ± 2.7^*^	88.8 ± 3.0	89.5 ± 1.9
AM	84.5 ± 4.2^*^	85.9 ± 3.4^*^	88.3 ± 2.8^*^	89.3 ± 2.3
EC	77.2 ± 7.1^*^	78.7 ± 5.1^*^	79.7 ± 4.7^*^	82.4 ± 4.2

*Ipsilateral/Contralateral refers to the epileptogenic hemisphere. Decreased performance relative to HybridMulti* -

* *: significant after Bonfferoni correction (p < 0.05/36 = 0.0013)*.

For the 3T data, even using a smaller dataset, we found that all the methods resulted in accuracy comparable to the larger 1.5T dataset, with generally decreased SDs. A separate test that segmented 3T dataset using the 1.5T training-set showed the result where we found overall a slight drop down in the accuracy and a larger SD (Controls: HP = 89.5 ± 2.4; AM = 89.0 ± 2.9; EC = 82.8 ± 4.4; TLE-ipsilateral: HP = 88.5 ± 2.8; AM = 89.1 ± 3.2; EC = 82.5 ± 4.9; TLE-contralateral: HP = 89.2 ± 2.6; AM = 89.1 ± 2.8; EC = 82.5 ± 5.2) compared to when using a smaller-set of the same field strength training data. This suggests that using a lower field training-set to segment a higher field strength data results in slightly decreased accuracy due to a different tissue-contrast.

Examples for 1.5T are shown in Figure 4 and those for 3T in Supplementary Figure 1.

#### Ability of automated methods to detect atrophy related to the epileptic focus

Group-wise comparisons identified hippocampal atrophy ipsilateral to the seizure focus in TLE patients irrespective of the method, i.e., manual or automated (*p* < 0.05, Table 3). The effect sizes of atrophy detected using algorithms were all large (Cohen's d > 0.8). HybridMulti and JointFusion, nevertheless, detected an effect size of atrophy closest to manual volumetry (Cohen's d: manual = 1.67; HybridMulti = 1.57; JointFusion = 1.56).

**Table 3:** Group differences between patients and controls.

	**Manual**	***HybridMulti***	**JointFusion**	**SurfMulti**	**Volmulti**
**HP**					
Ipsilateral	−**2.29** ± **1.85** (1.67)	−**2.09** ± **1.97** (1.58)	−**2.13** ± **2.37** (1.56)	−**1.69** ± **1.56** (1.39)	−**1.32** ± **1.62** (1.32)
Contralateral	−0.51 ± 1.70 (0.33)	−0.37 ± 1.53 (0.24)	−0.28 ± 1.78 (0.19)	−0.08 ± 1.34 (0.05)	−0.05 ± 1.45 (0.03)
**AM**					
Ipsilateral	−0.10 ± 1.45 (0.10)	−0.11 ± 1.41 (0.11)	0.39 ± 1.43 (−0.18)	0.05 ± 1.26 (−0.01)	0.35 ± 1.84 (−0.13)
Contralateral	0.20 ± 1.32 (−0.15)	0.27 ± 1.25 (−0.16)	0.45 ± 1.38 (−0.22)	0.15 ± 1.11 (−0.08)	−0.05 ± 1.71 (−0.02)
**EC**					
Ipsilateral	−**1.49** ± **1.19** (1.11)	−**0.98** ± **0.92** (0.82)	−0.52 ± 0.94 (0.45)	−0.65 ± 1.22 (0.46)	−0.35 ± 2.06 (0.18)
Contralateral	−0.69 ± 1.41 (0.52)	−0.37 ± 0.91 (0.29)	−0.18 ± 1.02 (0.16)	−0.15 ± 1.62 (0.09)	−0.05 ± 1.91 (0.03)

*Mesiotemporal volume in mean z-scores ± SD and effect sizes for atrophy shown in brackets (Cohen's d index; 0.2 indicates a small, 0.5 medium, and >0.8 large effect); group-wise significances in volumes (bold) are adjusted for multiple comparisons using Bonferroni correction*.

Manual and *HybridMulti* segmentation also detected a large effect size of ipsilateral EC atrophy, which was significant compared to controls (*t* > 3.2, *p* < 0.05).

#### Impact of template library size on segmentation accuracy

**Figure 5:**
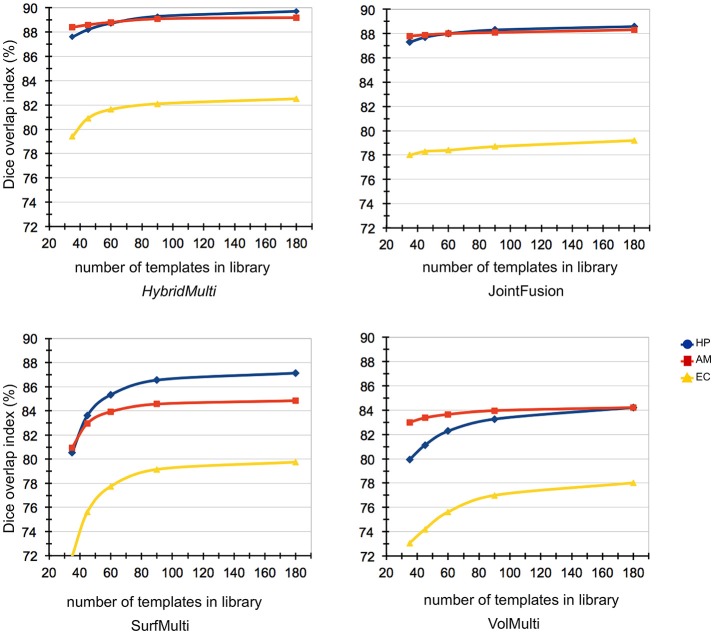
Impact of template library size on automated segmentations. Reducing the template library size from N (*n* = 175) to N/5 (*n* = 35) showed that the accuracy of EC segmentation declined fastest compared to HP and AM, consistently in all algorithms tested. Size of the library had a lower influence on segmentation accuracy of *HybridMulti*, and volume-based approaches (JointFusion, VolMulti) than SurfMulti.

Reducing the template library size from N (*n* = 175) to N/5 (*n* = 35) showed that the accuracy of EC segmentation declined fastest compared to HP and AM, consistently in all algorithms tested. Size of the library had a lower influence on segmentation accuracy of HybridMulti, and volume-based approaches (JointFusion, VolMulti) than SurfMulti. Indeed linear model analysis of an interaction term between “segmentation method” and “size of the library” revealed a faster decline in Dice index for SurfMulti than for the other three methods (*p* < 0.001). HybridMulti and JointFusion, on the other hand, resulted in a similar accuracy when reducing the template library size from N to N/4 across all MTL structures (mean Dice decrease < 1%, *p* < 0.1, Figure 5). In HP and EC, reducing the library size from N/4 to N/5 influenced the accuracy more significantly for HybridMulti than JointFusion (*p* < 0.01). However, the accuracy of HybridMulti was higher than that of JointFusion in all structures (mean Dice difference—HP: 0.3%; AM: 0.1%; E: 1%).

## Discussion and conclusion

We propose *HybridMulti*, an algorithm that combines surface- and volume-based similarity to automatically segment key regions in the mesiotemporal lobe (i.e., HP, AM, and EC). In controls and TLE patients alike, segmentation accuracy was excellent, with Dice indices above 88% for HP and AM and above 82% for EC. In particular, the proposed method outperformed previous multi-template approaches in pathological MTL structures, as its overlap to manual delineation and its sensitivity to detect atrophy were superior. Reducing template library showed that our method is reliable in even case of a small size of training-set.

Our algorithm was compared to three recently proposed multi-template approaches: volume-based approaches—JointFusion (Wang et al., 2013), VolMulti (Collins and Pruessner, 2010), and a purely surface-based framework—SurfMulti (Kim et al., 2012b). Improved segmentation accuracy of *HybridMulti* relative to these algorithms likely results from modeling both volume- and surface-derived features to select the optimal template subset and to improve the alignment between these templates and the test MRI prior to surface-shape averaging. Noticeably, our approach did not only sequentially apply a volumetric non-linear registration prior to the surface-based segmentation; instead, surface features were integrated with volume data-term into a unified cost function governing the non-linear registration, an approach yielding additional increases in accuracy.

In addition to absolute gain in segmentation accuracy, the proposed *HybridMulti* algorithm demonstrated robust segmentation for our two separate data-sets when the size of the template library was reduced, an important challenge for purely surface-based approaches as shown in our analysis. Indeed, volume-based approaches were inclined to maintain its original accuracy at the largest template library when reducing the size of the library. At the smallest size that was tested in our study (*n* = 35), the accuracy of JointFusion and *HybridMulti* was almost equal in all MTL structures. This informs us to an interesting aspect of feature modeling where local features modeled nearby the structure's boundary may be individually very specific and become powerful with construction of a large training-set. On the other hand, features collected within a “relatively large” volume of interest may include redundant information in a large database but may provide supplementary characteristics of the target structure in case of using a limited size of template library. In our hybrid approach, tuning of the weight between surface- and volume-features according to the size of a given template library can possibly improve the segmentation accuracy.

Our EC segmentation in the current work (>82%) outperformed a previous study that reported a Dice index of 73% (Hu et al., 2014), and another study that segmented the whole parahippocampal gyrus with a similar degree of accuracy (Heckemann et al., 2006). The performance of *HybridMulti* was also superior to JointFusion and SurfMulti in the current evaluation. Nevertheless, our EC segmentation accuracy was still lower than that of HP and AM, which approached 90%. It is likely that intensity-based segmentation is challenged by the highly variable morphology of the collateral sulcus that defines the border of EC. Also, the posterior end of EC is defined with an external anatomical landmark. Use of a smaller size of template library also showed a faster decline of accuracy in EC than other MTL structures. In the literature (Bernasconi et al., 2001; Pruessner et al., 2002), multiple landmarks were borrowed to address for lack of intensity contrast when defining the border of EC. For example, the medial and lateral boundaries, which meet the same GM structures such as the subiculum of the hippocampus and the collateral sulcus, cannot be defined by the tissue contrast but by landmarks such as a location with a high angular shape. A human expert may intuitively identify such landmarks whereas the features used in our algorithm do not necessarily take into account them. The suboptimal modeling of these landmarks in our approach is likely the source of inaccuracy in segmentation. This faster decline in accuracy was consistently observed in all algorithms tested. Future works might, therefore, benefit from the incorporation of sulco-gyral shape patterns such as sulcal depth, curvature or spatial relationship with surrounding structures other than HP and AM.

A new multi-scale weighting strategy improved EC and HP segmentation. In particular, the improvement of EC segmentation was higher. This was in line with a previous finding that such a technique mainly improve the segmentation of structures presenting highly variable morphology (Artaechevarria et al., 2009).

The proposed algorithm and JointFusion detected largest effect sizes of atrophy in HP ipsilateral to the epileptic focus and resulted in the most sensitivity to detect hippocampal atrophy among algorithms. Only *HybridMulti* identified EC atrophy among algorithm even if the accuracy yet is to reach human expert's exquisiteness. Our results suggest that the proposed approach may have the potential for clinical utility in the presurgical evaluation of temporal lobe epilepsy.

Varying the parameters for HybridMulti (i.e., the weights for surface-term in the similarity measure and the registration, and the number of templates in the subset) yielded different segmentation accuracy. We determined these parameters in empirical fashion for optimal segmentation performance. We observed that almost same parameter setting were determined for achievement of the best results on both 1.5T and 3T. In a further analysis, we found that these parameters did not differ between segmentation of the three MTL structures. This suggests that the parameters optimized in our study, albeit done empirically, may be generally applicable to segmentation of other datasets or other brain structures. A more thorough analysis is demanded to establish the generalization of the parameters.

For 3T dataset, all the methods resulted in accuracy comparable to the larger 1.5T dataset, with generally decreased SDs. This likely explains that reliable segmentation can be achieved on 3T images where the higher tissue contrast and clearer structural boundaries seen.

As the initial selection was not optimal and we did not like to miss templates which can be potentially useful, we defined a relatively lager subset whereas we set a smaller sample in the subsequent selection with a deformable registration. Our empirical selection of parameters indeed found better segmentation performance was obtained using a larger subset in the initial selection (best performance at *n* = 17) and a smaller set in the latter selection (*n* = 8). The vertex-wise correspondence between individual surface templates defined through SPHARM-PDM ensures the same topology across templates. When we averaged the template shapes, we performed a vertex-wise averaging method that averages the location of a given vertex of the correspondence between templates. The integrity of the topology was not corrupted after this averaging as the same observation is found in a similar process of shape averaging such as in construction of cortical surface template (Styner et al., 2004; Lyttelton et al., 2007).

To determine the number of templates with the best performance, it would be ideal if we observed a plateau occurring after the continuous hiking in Dice index value from the minimum number of templates to test with (Figure 5). However, no plateau with an on-going climbing pattern was found in EC, which make difficult to determine when the best performance takes place. The best performance might have been identified if we tested with more templates. This is our limitation as collecting a sufficiently large dataset is often a long-term procedure in the inpatient epilepsy monitoring unit. Thus, it was unrealistic for us to include more data in the study. Alternatively, the very slow increase in Dice index observed at the test with 90+ templates likely explains the increase of the templates would not gain a very significant improvement of the current method. There have been studies dealing with the size of the template library using statistical models (Awate et al., 2012; Awate and Whitaker, 2014).

We did not explore the possible selection of too many similar templates in the subset. A previous study (Wang et al., 2013) investigated this using a joint label fusion technique that address for the covariance of the image appearance between any pair of two templates in the training-set. Generalization of the proposed method across different subcortical structures (e.g., ventricles, striatum, or thalamic nucleus) would be also interesting to enable their morphometry analysis, in particular with regard to size, shape, and variability. We are also working on to extend our current framework to segmentation of the subregions of MTL structures such as hippocampal subfields. The deep learning algorithm using convolutional neural networks (CNN) has been more widely applied in recent works for the medical image segmentation (Kamnitsas et al., 2017; Bao and Chung, 2018; Dolz et al., 2018). Augmentation of our relatively large set of our MRI data and manual annotations could meet the requirement for the training of the CNNs, which can be a proper future extension of our work. We are currently taking steps to make our tools available, including obtaining proper institutional ethics approval, with the plan to ultimately upload the software and training set to a public domain, such as the Neuroimaging Informatics Tools and Resources Clearinghouse (http://www.nitrc.org/).

## Author contributions

HK implemented the study design and the algorithm. Performed the evaluation. Wrote and edited the draft. BC tested and re-tested the data using a conventional method to compare. AB provided the MRI data and patient clinical data. Edited the manuscript. NB manually labeled the MTL structures edited the manuscript.

### Conflict of interest statement

The authors declare that the research was conducted in the absence of any commercial or financial relationships that could be construed as a potential conflict of interest.

## References

[B1] Arnone D. Job D. Selvaraj S. Abe O. Amico F. Cheng Y.. (2016). Computational meta-analysis of statistical parametric maps in major depression. Hum. Brain Mapp. 37, 1393–1404. 10.1002/hbm.23108 26854015 PMC6867585

[B2] Artaechevarria X. Munoz-Barrutia A. Ortiz-de-Solorzano C. (2009). Combination strategies in multi-atlas image segmentation: application to brain MR data. IEEE Trans. Med. Imaging 28, 1266–1277. 10.1109/TMI.2009.2014372 19228554

[B3] Asman A. J. Landman B. A. (2013). Non-local statistical label fusion for multi-atlas segmentation. Med. Image Anal. 17, 194–208. 10.1016/j.media.2012.10.002 23265798 PMC3648421

[B4] Awate S. P. Whitaker R. T. (2014). Multiatlas segmentation as nonparametric regression. IEEE Trans. Med. Imaging 33, 1803–1817. 10.1109/TMI.2014.2321281 24802528 PMC4440593

[B5] Awate S. P. Zhu P. Whitaker R. T. (2012). How many templates does it take for a good segmentation?: Error analysis in multiatlas segmentation as a function of database size. Med. Image Comput. Comput. Assist. Interv. 7509, 103–114. 24501720 10.1007/978-3-642-33530-3_9 PMC3910563

[B6] Bao S. Q. Chung A. C. S. (2018). Multi-scale structured CNN with label consistency for brain MR image segmentation. Comput. Methods Biomech. Biomed. Eng. Imaging Vis. 6, 113–117. 10.1080/21681163.2016.1182072

[B7] Bernasconi N. Bernasconi A. Caramanos Z. Antel S. B. Andermann F. Arnold D. L. (2003). Mesial temporal damage in temporal lobe epilepsy: a volumetric MRI study of the hippocampus, amygdala and parahippocampal region. Brain 126, 462–469. 10.1093/brain/awg034 12538412

[B8] Bernasconi N. Bernasconi A. Caramanos Z. Dubeau F. Richardson J. Andermann F.. (2001). Entorhinal cortex atrophy in epilepsy patients exhibiting normal hippocampal volumes. Neurology 56, 1335–1339. 10.1212/WNL.56.10.1335 11376184

[B9] Bernhardt B. C. Kim H. Bernasconi N. (2013). Patterns of subregional mesiotemporal disease progression in temporal lobe epilepsy. Neurology 81, 1840–1847. 10.1212/01.wnl.0000436069.20513.92 24142475 PMC3821710

[B10] Cavedo E. Boccardi M. Ganzola R. Canu E. Beltramello A. Caltagirone C.. (2011). Local amygdala structural differences with 3T MRI in patients with Alzheimer disease. Neurology 76, 727–733. 10.1212/WNL.0b013e31820d62d9 21339500 PMC3053328

[B11] Collins D. L. Pruessner J. C. (2010). Towards accurate, automatic segmentation of the hippocampus and amygdala from MRI by augmenting ANIMAL with a template library and label fusion. Neuroimage 52, 1355–1366. 10.1016/j.neuroimage.2010.04.193 20441794

[B12] Collins D. L. Holmes C. J. Peters T. M. Evans A. C. (1995). Automatic 3-D model-based neuroanatomical segmentation. Hum. Brain Mapp. 3, 190–208. 10.1002/hbm.460030304

[B13] Collins D. L. Neelin P. Peters T. M. Evans A. C. (1994). Automatic 3D intersubject registration of MR volumetric data in standardized Talairach space. J. Comput. Assist. Tomogr. 18, 192–205. 10.1097/00004728-199403000-00005 8126267

[B14] Coupé P. Manjón J. V. Fonov V. Pruessner J. Robles M. Collins D. L. (2011). Patch-based segmentation using expert priors: application to hippocampus and ventricle segmentation. Neuroimage 54, 940–954. 10.1016/j.neuroimage.2010.09.018 20851199

[B15] Dolz J. Desrosiers C. Ben Ayed I. (2018). 3D fully convolutional networks for subcortical segmentation in MRI: a large-scale study. Neuroimage 170, 456–470. 10.1016/j.neuroimage.2017.04.039 28450139

[B16] Eskildsen S. F. Coupé P. Fonov V. Manjón J. V. Leung K. K. Guizard N.. (2012). BEaST: brain extraction based on nonlocal segmentation technique. Neuroimage 59, 2362–2373. 10.1016/j.neuroimage.2011.09.012 21945694

[B17] Goncharova I. I. Dickerson B. C. Stoub T. R. deToledo-Morrell L. (2001). MRI of human entorhinal cortex: a reliable protocol for volumetric measurement. Neurobiol. Aging 22, 737–745. 10.1016/S0197-4580(01)00270-6 11705633

[B18] Heckemann R. A. Hajnal J. V. Aljabar P. Rueckert D. Hammers A. (2006). Automatic anatomical brain MRI segmentation combining label propagation and decision fusion. Neuroimage 33, 115–126. 10.1016/j.neuroimage.2006.05.061 16860573

[B19] Hu S. Coupé P. Pruessner J. C. Collins D. L. (2014). Nonlocal regularization for active appearance model: application to medial temporal lobe segmentation. Hum. Brain Mapp. 35, 377–395. 10.1002/hbm.22183 22987811 PMC6869426

[B20] Joo E. Y. Kim H. Suh S. Hong S. B. (2014). Hippocampal substructural vulnerability to sleep disturbance and cognitive impairment in patients with chronic primary insomnia: magnetic resonance imaging morphometry. Sleep 37, 1189–1198. 10.5665/sleep.3836 25061247 PMC4098804

[B21] Kamnitsas K. Ledig C. Newcombe V. F. J. Simpson J. P. Kane A. D. Menon D. K.. (2017). Efficient multi-scale 3D CNN with fully connected CRF for accurate brain lesion segmentation. Med. Image Anal. 36, 61–78. 10.1016/j.media.2016.10.004 27865153

[B22] Keihaninejad S. Heckemann R. A. Gousias I. S. Hajnal J. V. Duncan J. S. Aljabar P.. (2012). Classification and lateralization of temporal lobe epilepsies with and without hippocampal atrophy based on whole-brain automatic MRI segmentation. PLoS ONE 7:e33096. 10.1371/journal.pone.0033096 22523539 PMC3327701

[B23] Khan A. R. Cherbuin N. Wen W. Anstey K. J. Sachdev P. Beg M. F. (2011). Optimal weights for local multi-atlas fusion using supervised learning and dynamic information (SuperDyn): validation on hippocampus segmentation. Neuroimage 56, 126–139. 10.1016/j.neuroimage.2011.01.078 21296166

[B24] Kim H. Chupin M. Colliot O. Bernhardt B. C. Bernasconi N. Bernasconi A. (2012a). Automatic hippocampal segmentation in temporal lobe epilepsy: impact of developmental abnormalities. Neuroimage 59, 3178–3186. 10.1016/j.neuroimage.2011.11.040 22155377

[B25] Kim H. Mansi T. Bernasconi N. Bernasconi A. (2012b). Surface-based multi-template automated hippocampal segmentation: application to temporal lobe epilepsy. Med. Image Anal. 16, 1445–1455. 10.1016/j.media.2012.04.008 22613821

[B26] Lagarias J. C. Reeds J. A. Wright M. H. Wright P. E. (1998). Convergence properties of the Nelder-Mead simplex method in low dimensions. Siam J. Optimiz. 9, 112–147. 10.1137/S1052623496303470

[B27] Landman B. Warfield S. (2012). MICCAI 2012 workshop on multi-atlas labeling, in Proc. Med. Image Comput. Comput. Assisted Intervent. Conf. Grand Challenge Workshop Multi-Atlas Labeling Challenge Result (Nice).

[B28] Lyttelton O. Boucher M. Robbins S. Evans A. (2007). An unbiased iterative group registration template for cortical surface analysis. Neuroimage 34, 1535–1544. 10.1016/j.neuroimage.2006.10.041 17188895

[B29] Maccotta L. Moseley E. D. Benzinger T. L. Hogan R. E. (2015). Beyond the CA1 subfield: local hippocampal shape changes in MRI-negative temporal lobe epilepsy. Epilepsia 56, 780–788. 10.1111/epi.12955 25809286 PMC4527133

[B30] Pruessner J. C. Kohler S. Crane J. Pruessner M. Lord C. Byrne A.. (2002). Volumetry of temporopolar, perirhinal, entorhinal and parahippocampal cortex from high-resolution MR images: considering the variability of the collateral sulcus. Cereb. Cortex 12, 1342–1353. 10.1093/cercor/12.12.1342 12427684

[B31] Schramm J. Clusmann H. (2008). The surgery of epilepsy. Neurosurgery 62(Suppl. 2), 463–481; discussion 481. 10.1227/01.neu.0000316250.69898.23 18596456

[B32] Shi J. Thompson P. M. Gutman B. Wang Y. Alzheimer's Disease Neuroimaging, I. (2013). Surface fluid registration of conformal representation: application to detect disease burden and genetic influence on hippocampus. Neuroimage 78, 111–134. 10.1016/j.neuroimage.2013.04.018 23587689 PMC3683848

[B33] Shoemake K. (1985). Animating rotation with quaternion curves. SIGGRAPH Comput. Graph. 19, 245–254. 10.1145/325165.325242

[B34] Sled J. G. Zijdenbos A. P. Evans A. C. (1998). A nonparametric method for automatic correction of intensity nonuniformity in MRI data. IEEE Trans. Med. Imaging 17, 87–97. 10.1109/42.668698 9617910

[B35] Studholme C. Hill D. L. G. Hawkes D. J. (1999). An overlap invariant entropy measure of 3D medical image alignment. Pattern Recognit. 32, 71–86. 10.1016/S0031-3203(98)00091-0

[B36] Styner M. Lieberman J. A. Pantazis D. Gerig G. (2004). Boundary and medial shape analysis of the hippocampus in schizophrenia. Med. Image Anal. 8, 197–203. 10.1016/j.media.2004.06.004 15450215

[B37] Styner M. Oguz I. Xu S. Brechbühler C. Pantazis D. Gerig G. (2006a). Statistical shape analysis of brain structures using SPHARM-PDM, in MICCAI 2006 Opensource Workshop (Copenhagen). PMC3062073 21941375

[B38] Styner M. Oguz I. Xu S. Brechbuhler C. Pantazis D. Levitt J. J.. (2006b). Framework for the statistical shape analysis of brain structures using SPHARM-PDM. Insight J. 242–250. 21941375 PMC3062073

[B39] Wang H. Z. Suh J. W. Das S. R. Pluta J. B. Craige C. Yushkevich P. A. (2013). Multi-atlas segmentation with joint label fusion. IEEE Trans. Pattern Anal. Mach. Intell. 35, 611–623. 10.1109/TPAMI.2012.143 22732662 PMC3864549

[B40] Wang Z. Neylan T. C. Mueller S. G. Lenoci M. Truran D. Marmar C. R.. (2010). Magnetic resonance imaging of hippocampal subfields in posttraumatic stress disorder. Arch. Gen. Psychiatry 67, 296–303. 10.1001/archgenpsychiatry.2009.205 20194830 PMC2848481

