# Supplementary figures and images for: Multi-Template Mesiotemporal Lobe Segmentation: Effects of Surface and Volume Feature Modeling
Source: Front Neuroinform. 2018 Jul 12;12:39. doi: 10.3389/fninf.2018.00039 (PMC6052096; doi:10.3389/fninf.2018.00039)

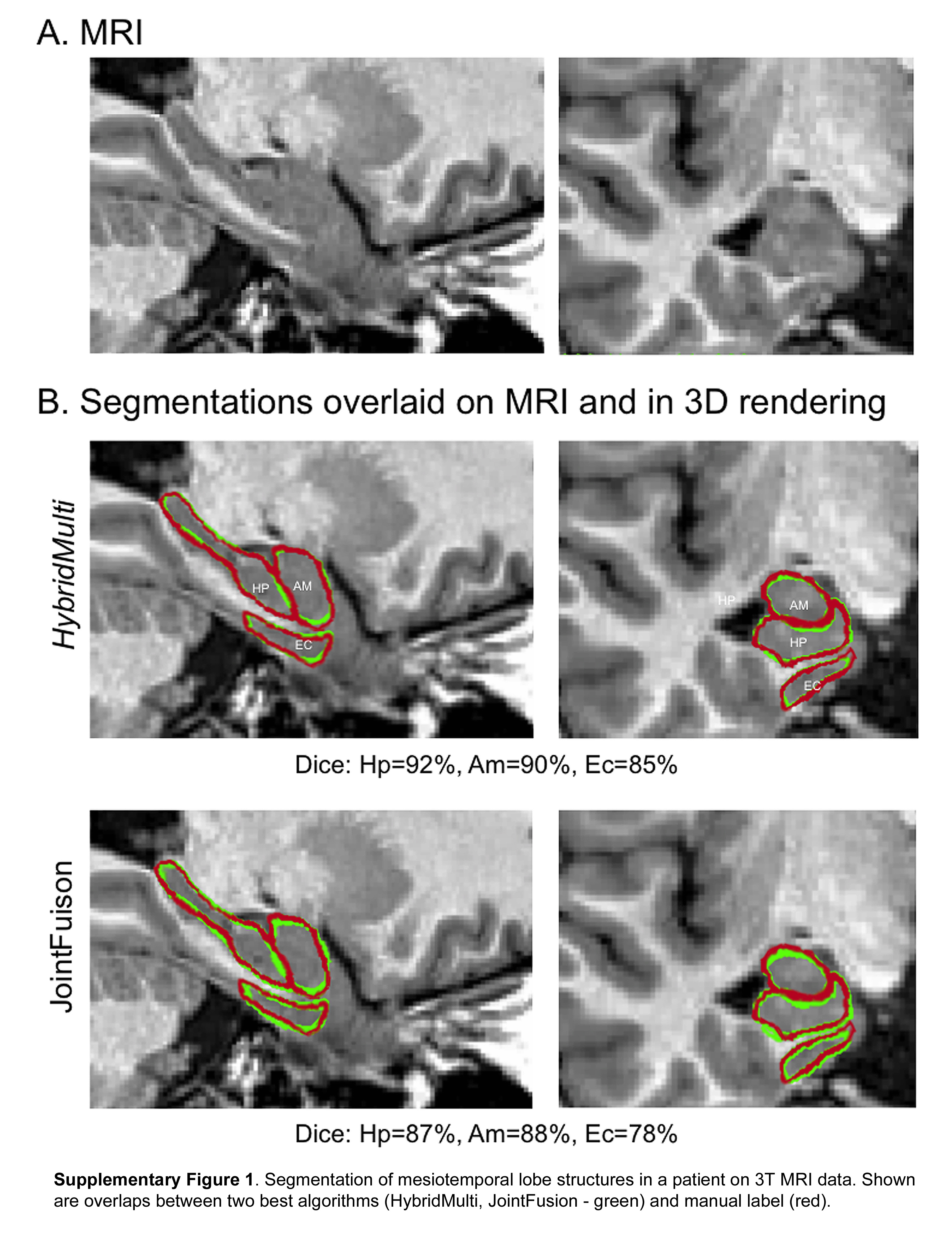

Supplement: Supplementary file 1 [file Image_1.jpg]
